# Comparison of pathogenic and non-pathogenic *Enterococcus cecorum* strains from different animal species

**DOI:** 10.1186/s12866-017-0949-y

**Published:** 2017-02-13

**Authors:** Arne Jung, Martin Metzner, Martin Ryll

**Affiliations:** 1 Clinic for Poultry, University of Veterinary Medicine Hannover, Buenteweg 17, D-30559 Hannover, Germany; 2 RIPAC LABOR GmbH, Am Muehlenberg 11, D-14476 Potsdam-Golm, Germany

**Keywords:** Enterococcus cecorum, Virulence factors, Poultry, Chicken embryo lethality assay, Fatty acid profile, MALDI-TOF mass spectrometry, Serotyping

## Abstract

**Background:**

*Enterococcus cecorum* (EC) infection currently is one of the most important bacterial diseases of modern broiler chickens but can also affect ducks or other avian species. However, little is known concerning pathogenesis of EC and most studies concentrate on examinations of EC strains from broilers only. The objective of this study was to compare pathogenic and commensal EC strains from different animal species concerning different phenotypic and genotypic traits.

**Results:**

Pathogenic and commensal EC strains were not clearly separated from each other in a phylogenetic tree based on partial sequences of the 16S-rRNA-gene and also based on the fatty acid profile determined with gas chromatography. C_12:0_, C_14:0_, C_15:0_, C_16:0_, C_17:0_, C_18:0_, C_18:1_ w7c, C_18:1_ w9c and C_20:4_ w6,9,12,15c were detected as the major fatty acids. None of the 21 pathogenic EC strains was able to utilize mannitol, while 9 of 29 commensal strains were mannitol positive. In a dendrogram based on MALDI-TOF MS data, pathogenic strains were not clearly separated from commensal isolates. However, significant differences concerning the prevalence of several mass peaks were confirmed between the two groups. Two different antisera were produced but none of the serotypes was predominantly found in the pathogenic or commensal EC isolates. Enterococcal virulence factors *gelE*, *esp*, *asa1*, *ccf*, *hyl* and *efaAfs* were only detected in single isolates via PCR. No virulence factor was found significantly more often in the pathogenic isolates. The chicken embryo lethality of the examined EC isolates varied from 0 up to 100%. The mean embryo lethality in the pathogenic EC isolates was 39.7%, which was significantly higher than the lethality of the commensal strains, which was 18.9%. Additionally, five of the commensal isolates showed small colony variant growth, which was never reported for EC before.

**Conclusions:**

Pathogenic and commensal EC isolates from different animal species varied in chicken embryo lethality, in their ability to metabolize mannitol and probably showed divergent mass peak patterns with MALDI-TOF MS. These differences may be explained by a separate evolution of pathogenic EC isolates. Furthermore, different serotypes of EC were demonstrated for the first time.

## Background

*Enterococcus cecorum* (EC) can be designated as an emerging avian pathogen. In the last 10 years it became one of the most important bacterial diseases in commercial broiler operations and outbreaks were reported from Belgium [1], Germany [2], Hungary [3] the Netherlands [4, 5], Poland [6], Scotland [7], Switzerland [8], Canada [9], the United States [10] and recently also from South Africa and Malaysia [11, 12]. EC infected broiler flocks usually develop clinical signs between 5 to 10 weeks of age with a marked increase in flock mortality [10, 13]. Affected birds exhibit spondylitis of the free thoracic vertebra and lesions in the femoral head that are consistent with the clinical diseases known as femoral head necrosis or bacterial chondritis and osteomyelitis. The spinal or hip lesions cause lameness and hind-limb paresis. EC associated disease outbreaks were also reported from ducks in Germany [14, 15] and EC infection was successfully reproduced experimentally in Pekin ducks [16]. Additionally, septicemic EC infection was described in single pigeons [17, 18], demonstrating the ability of EC to induce disease in other birds than chickens. EC infections in humans were also sporadically reported, but with an increasing frequency, predominantly as hospital-acquired infections [19–27]. On the other hand, EC is a member of the physiological microbiota of the intestine of chickens [28, 29] and has been isolated from the intestinal tract of healthy horses, cattle, pigs, dogs, cats, chickens, canaries, pigeons, turkeys and Muscovy ducks [28, 30–33]. So far, little is known about the pathogenesis of EC infection and the properties of EC which influence its pathogenic potential. Additionally, no data is available regarding the virulence of EC isolates recovered from animals other than broiler chickens. In this study virulence and different phenotypic and genotypic properties of pathogenic and commensal *Enterococcus cecorum* strains from different animal species were compared.

## Methods

### Bacterial strains

EC strains were obtained from samples submitted for diagnostic procedures. The strains were isolated from 1995 to 2015. All strains are listed in Table 1 including isolate number and animal species. Strains were classified into the 2 categories “pathogenic” and “commensal” based on source of isolation (organs from diseased vs. intestine from healthy animals) and presence of clinical signs and/or pathological changes. Strains were archived as pure cultures using the cryobank system (Mast Diagnostica GmbH, Reinfeld, Germany). The EC type strain DSM 20682 was obtained from Deutsche Sammlung von Mikroorganismen und Zellkulturen GmbH (Braunschweig, Germany).

**Table 1:** *Enterococcus cecorum* isolates included in this study, their phenotypic and genotypic properties, chicken embryo lethality and serotype

PCR results for virulence genes														
Isolate number	Animal species/production type	Classification of isolate^b^	Colony morpholgy	D-mannitol metabolism	Cytolysin (*cylA*)	Enterococcal surface protein (*esp*)	Aggregation substance (*asa1*)	Hyaluronidase (*hyl*)	Gelatinase (*gelE*)	Cell wall adhesin of *E. faecium* (*efaAfm*)	Cell wall adhesin of *E. faecalis* (*efaAfs*)	Sex pheromone (*ccf*)	Chicken embryo lethality in %^e^	Serotype^f^
x288/5-95	Broiler	Commensal	SCV^c^	-	-	-	+	-	-	-	-	-	20.0	n.t.^g^
09/309/1/A	Broiler	Pathogenic	N^d^	-	-	-	-	-	-	-	-	-	0.0	n.t.
09/310/1/A	Broiler	Pathogenic	N	-	-	+	-	+	+	-	-	-	60.0	n.t.
12/673/4/A	Broiler	Pathogenic	N	-	-	-	-	-	-	-	-	-	6.7	n.t.
13/655/2/B	Broiler	Commensal	N	-	-	+	+	-	+	-	+	-	100.0	n.t.
13/655/3/B	Broiler	Commensal	N	-	-	-	-	-	+	-	-	-	25.0	n.t.
14/086/4/A	Broiler	Pathogenic	N	-	-	-	-	-	-	-	-	-	86.7	n.t.
14/086/9/A	Broiler	Pathogenic	N	-	-	-	-	-	-	-	-	-	53.3	n.t.
14/093/1/A	Broiler	Pathogenic	N	-	-	-	-	-	-	-	-	-	35.7	n.t.
14/166/2/A	Broiler	Pathogenic	N	-	-	-	-	-	-	-	-	-	80.0	2
14/359/1/A	Broiler	Pathogenic	N	-	-	-	-	-	-	-	-	-	33.3	n.t.
15/218/1/A	Broiler	Pathogenic	N	-	-	-	-	-	+	-	-	-	6.7	2
15/827/1/A	Broiler	Pathogenic	N	-	-	-	-	-	-	-	-	-	80.0	2
15/828/1/A	Broiler	Pathogenic	N	-	-	-	-	-	-	-	-	-	46.2	1
15/839/1/A	Broiler	Pathogenic	N	-	-	-	-	-	-	-	-	-	46.7	2
x829/5c-01	Pekin duck	Commensal	SCV	-	-	-	-	-	-	-	-	-	0.0	2
x1610/1-01	Pekin duck	Commensal	SCV	-	-	-	-	-	-	-	-	-	0.0	n.t.
10/063/20/A	Pekin duck	Pathogenic	N	-	-	+	-	+	-	-	-	-	53.3	2
13/698/3/B	Pekin duck	Commensal	N	-	-	+	-	-	-	-	-	-	8.3	2
14/456/2/A	Pekin duck	Commensal	N	-	-	-	-	-	-	-	-	-	13.3	n.t.
D07-797-90/61	Pekin duck	Pathogenic	N	-	-	+	-	+	+	-	-	-	60.0	1
D09-367-6	Pekin duck	Commensal	N	-	-	-	-	-	-	-	-	-	0.0	n.t.
D10-455-1	Pekin duck	Pathogenic	N	-	-	-	-	-	-	-	-	-	46.6	2
D11-0675-2-8-3	Pekin duck	Pathogenic	N	-	-	-	-	-	-	-	-	-	0.0	n.t.
D12-0364-1-1-1	Pekin duck	Pathogenic	N	-	-	-	-	-	-	-	-	-	64.3	n.t.
D14-2291-5-7-1	Pekin duck	Pathogenic	N	-	-	-	-	-	-	-	-	-	0.0	2
x204/2b-95	Laying hen	Commensal	N	-	-	-	-	-	-	-	-	-	20.0	n.t.
x242/3-96	Laying hen	Commensal	SCV	-	-	-	+	-	-	-	-	-	0.0	2
12/284/4/A	Laying hen	Commensal	N	-	-	+	-	-	+	-	-	-	6.7	n.t.
13/428/1/A	Laying hen	Commensal	N	+	-	-	-	-	-	-	-	-	28.6	n.t.
13/429/1/A	Laying hen	Commensal	N	+	-	-	-	-	-	-	-	-	46.6	n.t.
15/308/6/D	Laying hen	Commensal	N	-	-	-	-	-	-	-	-	-	0.0	n.t.
x150/2-95	Turkey	Commensal	N	-	-	-	-	-	-	-	-	-	0.0	2
D11-0325-3-1-2	Turkey	Pathogenic	N	-	-	-	-	-	-	-	-	-	20.0	1
D11-1088-2-1-1	Turkey	Commensal	N	-	-	-	-	-	-	-	-	-	33.3	n.t.
D12-0108-3-3-3	Turkey	Commensal	N	-	-	-	-	-	-	-	-	-	13.3	n.t.
D13-0112-3-1-2	Turkey	Commensal	N	-	-	-	-	-	-	-	-	-	13.3	1
x459/1-95	Pigeon	Commensal	N	+	-	-	+	-	-	-	-	+	0.0	1
14/022/1/A	Pigeon	Commensal	N	-	-	-	-	-	-	-	-	-	0.0	1
D12-0869-2-1-1	Pigeon	Commensal	N	+	-	-	-	-	-	-	-	-	0.0	1
D14-1051-5-1-4	Pigeon	Commensal	N	-	-	-	-	-	-	-	-	-	6.7	n.t.
D12-0239-4-1-2	Cattle	Commensal	N	+	-	-	-	-	-	-	-	-	66.7	1
D12-1662-1-14-1	Cattle	Commensal	N	+	-	-	-	-	-	-	-	-	20.0	n.t.
D11-0174-10-1-3	Swine	Commensal	N	+	-	-	-	-	-	-	-	-	93.3	n.t.
D13-0364-1-1-4	Swine	Commensal	N	+	-	-	-	-	+	-	-	-	13.3	2
x23/1-95	Budgerigar	Commensal	N	+	-	-	-	-	-	-	-	+	13.3	2
12/437/1/C	Goose	Commensal	N	-	-	-	-	-	+	-	-	+	6.7	n.t.
14/1103/1/A	Human	Pathogenic	N	-	-	-	-	-	-	-	-	-	13.3	n.t.
10/704/4/A	Muscovy duck	Pathogenic	N	-	-	-	-	-	-	-	+	+	40.0	n.t.
x495/3-95	Swan	Commensal	SCV	-	-	-	-	-	-	-	-	-	0.0	1
*DSM 20682* ^*a*^	*Chicken*	*Commensal*	*N*	-	-	+	-	+	-	-	-	-	*63.6*	*n.t.*

^a^Type strain; other strain collection numbers: ATCC 43198, NCDO 2674. ^b^Based on source of isolation and presence of clinical signs/pathology. ^c^Small colony variant strains of *Enterococcus cecorum*. ^d^Normal colony morphology. ^e^15 chicken eggs per isolate were inoculated with 10^2^ CFU/egg into the allantoic cavity and examined for embryonic death for 7 days post inoculation. ^f^Two different antisera were produced from field strains D07-797-90/61 (Pekin duck, serotype 1) and 15/827/1/A (broiler, serotype 2) and isolates were tested using the slide agglutination procedure. ^g^Non-typeable with the two produced antisera

### Bacterial cultivation

For the evaluation of the colony morphology, EC strains were cultivated on Columbia sheep blood (CSB) agar plates (Oxoid, Wesel, Germany) and incubated for 24 h at 37 °C in a CO_2_-enriched atmosphere (5% CO_2_).

#### 16S-rRNA-gene sequencing

Bacterial DNA of the EC strains was isolated using the boiling method. Briefly, pure subcultures were produced of each strain and one loop culture material was mixed into 500 μl molecular biology grade water. Samples were incubated at 95 °C and 500 rpm in a heating bloc with shaking function and centrifuged at 13.000 × g for 5 min. The supernatant was archived at -20 °C. A 440-bp segment of the 16S-rRNA gene was amplified using primers 91E-for (GGAATTCAAAKGAATTGACGGGGGC) and 13B-rev (CG GGATCCCAGGCCCGGGAACGTATTCAC) [34]. For single strains, the 440 bp segment was too short to allow reliable identification. In these cases, different primer sets and PCR conditions were used for identification [35, 36], producing amplificates of 1502 or 721 bp respectively. PCR products were sequenced at Microsynth AG (Lindau, Germany). DNA sequence analysis was performed using the BLAST database of the American National Center for Biotechnology Information (Bethesda, Maryland, USA) and the EzTaxon server [37], which contains only type strains. Phylogenetic analysis of the 16S-rRNA-gene sequences was done with MEGA7 [38] using neighbor joining method with Euclidean distances.

### MALDI-TOF sample preparation and MS analysis

EC was identified by means of “Matrix-assisted linear desorption/ionization time-of-flight mass spectrometry” (MALDI-TOF MS) technique (VITEK MS RUO, bioMerieux Deutschland GmbH; formally AXIMA@SARAMIS) as described before [39]. The strains were analyzed on a MAB-AG stainless steel target (MAB-AG, Switzerland), using a whole-cell protocol with 1 μl matrix solution of saturated α-cyano-4 hydroxy-cinnamic acid in a mixture of acetonitrile, ethanol, and water (1:1:1) acidified with 3% (v/v) trifluoroacetic acid. For each strain, mass spectra were prepared in duplicate and analyzed in the linear positive ion extraction mode. Mass spectra were accumulated from 100 profiles, each from five nitrogen laser pulse cycles, by scanning the entire sample spot. Ions were accelerated with pulsed extraction at a voltage of 20 kV. Raw mass spectra were processed automatically for baseline correction and peak recognition. Resulting mass fingerprints were exported to the SARAMIS (Spectral Archiving and Microbial Identification System, AnagnosTec GmbH, Potsdam, Germany) analysis program and compared to reference superspectra and spectra to identify the species. Comparison of isolates was also carried out by SARAMIS software using the single link cluster algorithm and results are displayed in dendrograms showing Euclidean distances.

### Detection of the fatty acid composition by gas chromatography

Bacterial isolates were subcultured on CSB agar plates using the quadrant streak pattern and grown for 24 h at 35 °C. Bacteria in quadrant 3 (approximately 40 mg) were harvested. Cellular fatty acids were extracted and transformed into fatty acid methyl esters (FAMEs) according to the procedures described in the Sherlock Microbial Identification System (MIS) operating manual (Version 4.0, MIDI Microbial IDentification Inc, Delaware, USA). After the extraction step, FAMEs were injected into a HP 5890A gas chromatograph equipped with an automatic injector (injector HP 7673), sample controller, a gas chromatograph capillary column (Ultra 2, crosslinked 5% phenyl methyl silicone, 25 m × 0.2 mm × 0.11 μm film thickness) and a flame ionization detector (FID). Fatty acids with up to 20 carbon atoms (C-9 – C-20) were measured in a hydrogen phase. A calibration mix (Hewlett Packard) was included in every run as an internal reference. For data analysis, the CLIN40 method (Sherlock MIS Version 4.0, Microbial IDentification Inc, Delaware, USA) was used. A library validation report was generated by the MIDI procedures using the Sherlock MIS operating system. A dendrogram showing the phylogenetic relationship of the strains based on their fatty acid profile was generated by the same system via calculation with the UPGMA method (Unweighted Pair Group Method with Arithmetic Mean) using Euclidean distances.

### Mannitol metabolism

D-mannitol metabolism of the EC strains was evaluated as follows: one inoculation loop pure culture material was gently mixed into 5 ml of mannitol suspension (Merck KGaA, Darmstadt, Germany) and inoculated at 37 °C in a CO_2_-enriched atmosphere (5% CO_2_). Suspensions were evaluated after 48 h according to the color changes, yellow suspensions were considered positive, pink suspensions as negative.

### Serotyping

Serotypes of selected EC field isolates were determined by slide agglutination. A set of two anti-EC reference sera was used. Those polyclonal antisera were produced in rabbits by hyper-immunization according to a three months protocol with inactivated field strains D07_797-90/61 (Pekin duck, serotype 1) and 15/827/1/A (broiler, serotype 2) which are included in this study (see Table 1). Well grown single colonies of cultures grown at 37 °C under microaerophilic conditions for 24 h were homogenized by steel loop on a glass slide with 15 μl of serum. Only agglutination observed within 1 min in the reaction with each serum was regarded as positive. Delayed agglutination >1 min was regarded as negative.

### Detection of virulence factors

EC isolates were tested with classical polymerase chain reaction (PCR) for the enterococcal virulence factors cytolysin (*cylA*), enterococcal surface protein (*esp*), aggregation substance (*asa1*), hyaluronidase (*hyl*), gelatinase (*gelE*) [40], cell wall adhesins of *Enterococcus faecium* (*efaAfm*), cell wall adhesins of *Enterococcus faecalis* (*efaAfs*) and sex pheromone (*ccf*) [41]. Bacterial DNA was isolated using a commercially available mini spin filter system (innuPrep bacteria DNA kit; Analytic Jena, Jena, Germany). Virulence genes were detected using single PCRs and the HotStarTaq Plus Master Mix Kit (Qiagen, Hilden, Germany) in a final volume of 25 μl reaction mix. One reaction mix contained 9.5 μl of RNase-free water, 12.5 μl of HotStarTaq Plus Master Mix 2x, 0.5 μl forward and reverse primers (10pmol/μl) and 2 μl of template DNA respectively. The PCR was conducted using a SensoQuest labcycler (SensoQuest, Göttingen, Germany) with the following temperature profile: one cycle at 95 °C for 5 min followed by 40 cycles at 95 °C for 30 s, 55 °C for 90 s and 72 °C for 90 s. The final elongation step was performed for 10 min at 72 °C. PCR products were separated on 2% agarose gels and examined for the specific fragment sizes (Table 2). *Entercoccus faecalis* strain MMH595 and *Enterococcus faecium* strain C68 [40] served as positive controls for the 8 virulence factors.

**Table 2:** *Enterococcus* virulence genes and PCR primers which were used in this study

Virulence factor	Gene	Localisation	Primer name	Oligonucleotide sequence (5’ to 3’)	Product size	Primer source
Cytolysin	*cylA*	Chromosome/Plasmide	CYT I CYT IIb	ACTCGGGGATTGATAGGC GCTGCTAAAGCTGCGCTT	688	Vankerckhoven et al., 2004 [40]
Enterococcal surface protein	*esp*	Chromosome	ESP 14 F ESP 12R	AGATTTCATCTTTGATTCTTGG AATTGATTCTTTAGCATCTGG	510	Vankerckhoven et al., 2004 [40]
Hyaluronidase	*hyl*	Chromosome	HYL n1 HYL n2	ACAGAAGAGCTGCAGGAAATG GACTGACGTCCAAGTTTCCAA	276	Vankerckhoven et al., 2004 [40]
Aggregation substance	*asa1*	Plasmide	ASA 11 ASA 12	GCACGCTATTACGAACTATGA TAAGAAAGAACATCACCACGA	375	Vankerckhoven et al., 2004 [40]
Gelatinase	*gelE*	Chromosome	GEL11 GEL12	TATGACAATGCTTTTTGGGAT AGATGCACCCGAAATAATATA	213	Vankerckhoven et al., 2004 [40]
Cell wall adhesin of *E. faecium*	*efaAfm*	Chromosome	TE37 TE38	AACAGATCCGCATGAATA CATTTCATCATCTGATAGTA	735	Reviriego et al., 2005 [41]
Cell wall adhesin of *E. faecalis*	*efaAfs*	Chromosome	TE5 TE6	GACAGACCCTCACGAATA AGTTCATCATGCTGTAGTA	705	Reviriego et al., 2005 [41]
Sex pheromone	*ccf*	Chromosome	TE53 TE54	GGGAATTGAGTAGTGAAGAAG AGCCGCTAAAATCGGTAAAAT	543	Reviriego et al., 2005 [41]

### Chicken embryo lethality assay

Fresh subcultures of EC strains were prepared on CSB agar and incubated for 18 h at 37 °C and microaerophilic conditions. EC suspensions were prepared in physiological NaCl with the previously determined McFarland Standard of 6.9 (corresponds to 10^9^ CFU/ml) using the Densimat (Biomerieux, Marcy l’Etoile, France). A 10-fold dilution series was performed and the dilution of about 10^3^ CFU/ml was used for inoculation. SPF layer type chicken eggs (Valo Biomedia, Osterholz-Scharmbeck, Germany) were incubated at 37.5 °C and 50–60% humidity for 10 days and candled, infertile or non-viable eggs were removed before inoculation. Eggs were labeled with the isolate number and the blunt end with the air chamber was disinfected with 70% isopropanol. A hole was made in each shell using a metal drill. Fifteen eggs for each EC isolate were inoculated with 0.1 ml EC suspension (about 10^2^ CFU/egg) via the allantoic cavity using 0.70 × 30 mm needles and 1 ml syringes. Fifteen control eggs were inoculated with 0.1 ml physiological NaCl. The holes in the shells were sealed with glue. All eggs were incubated for 7 days post inoculation and candled daily. All dead embryos were counted for each isolate. Dead eggs from one and two days post infection were examined and eggs with vascular damage caused by the injection needle were excluded from the lethality calculation. All surviving embryos were sacrificed by decapitation at day 17 of incubation. Selected eggs were opened and sampled for reisolation of EC. EC suspensions which were used for inoculation were processed for 10-fold dilution series and calculation of CFU.

### Statistical analysis

The prevalence of each serotype in pathogenic and commensal strains was compared using the Chi-Square test. The prevalence of single masses from the MALDI-TOF MS and single virulence factors in pathogenic and commensal strains was compared using Fisher’s exact test. Pathogenic and commensal EC isolates were also analyzed concerning differences of the variable “chicken embryo letality”. First, Shapiro-Wilk test was used to test the data for normal distribution. As data was not normally distributed, Kruskal-Wallis test and post hoc test Dunn’s All-Pairwise Comparisons was selected for further comparisons. All calculations were done using Statistix 10 (Analytical Software, Tallahassee, Florida, USA). Differences in all statistical tests were considered significant at *P* ≤ 0.05.

## Results

### Bacterial cultivation

**Fig. 1:**
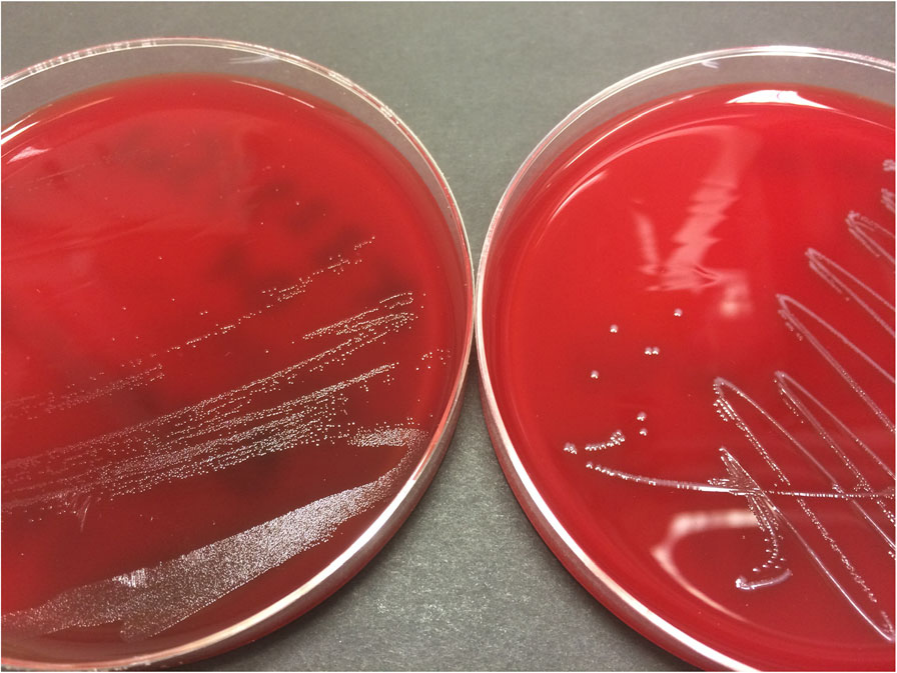
Colony growth of EC on Columbia sheep blood agar plates after 24 h incubation at 37 °C under microaerophilic conditions. The *left plate* shows small colony variant strain x829/5c-01, the *right plate* show strain D11-1088-2-1-1 with normal colony morphology

**Fig. 2:**
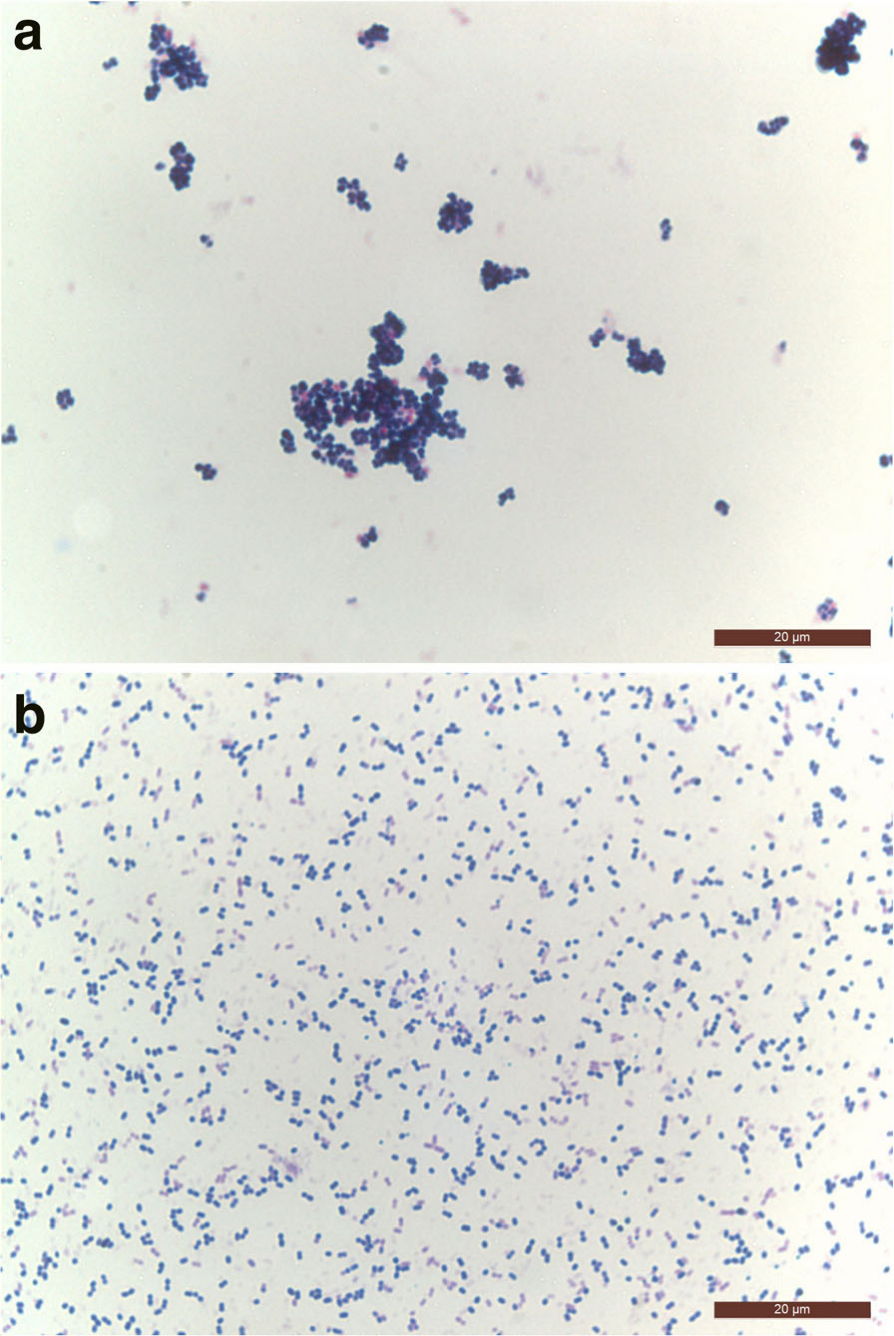
**a**. Gram staining of EC small colony variant strain x829/5c-01 showing conglomerates of cocci with very heterogeneous sizes and shapes. **b**. Gram staining of EC strain D11-1088-2-1-1 with normal colony morphology; both 1000 fold magnification, scale bar 20 μm

After 24 h, most of the isolates had grown as grey-white colonies with a diameter of 2–3 mm and weak α-hemolysis. Interestingly, five strains developed only small colonies <1 mm (Fig. 1). By Gram staining, these strains consisted of cocci with very heterogeneous sizes and shapes, including conglomerates of large cocci up to 2 μm in diameter (Fig. 2a). In contrast, strains with normal colony morphology showed regular small cocci which were homogeneously distributed (Fig. 2b). The five small colony variant (SCV) strains originated from broiler, Pekin duck (two isolates), laying hen and swan. All SCV isolates were classified as commensal.

### 16S-rRNA-gene sequencing

**Fig. 3:**
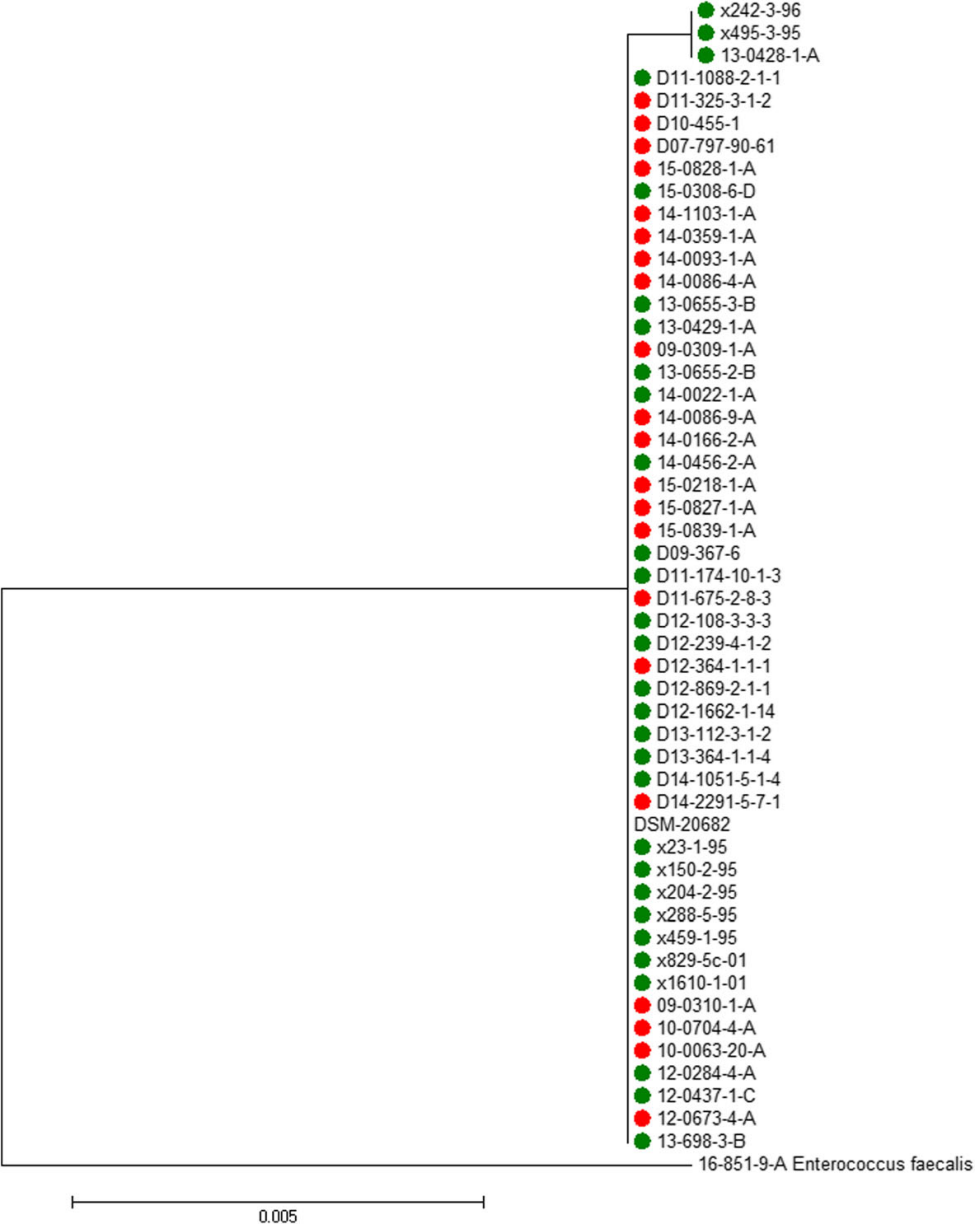
Dendrogram based on a 411 bp segment of the 16S rRNA gene of 50 EC isolates plus the reference strain DSM 20682. Pathogenic strains are labeled with in *red*, commensal strains in *green*

All isolates were confirmed as EC using 16S-rRNA-gene-sequencing. Sequences are available in GenBank under accession numbers KX674309-KX674359. In a phylogenetic tree based on the 16S-rRNA-gene sequences most pathogenic and commensal isolates formed a single cluster, while a small subcluster contained three commensal strains (Fig. 3).

### MALDI-TOF MS analysis

**Fig. 4:**
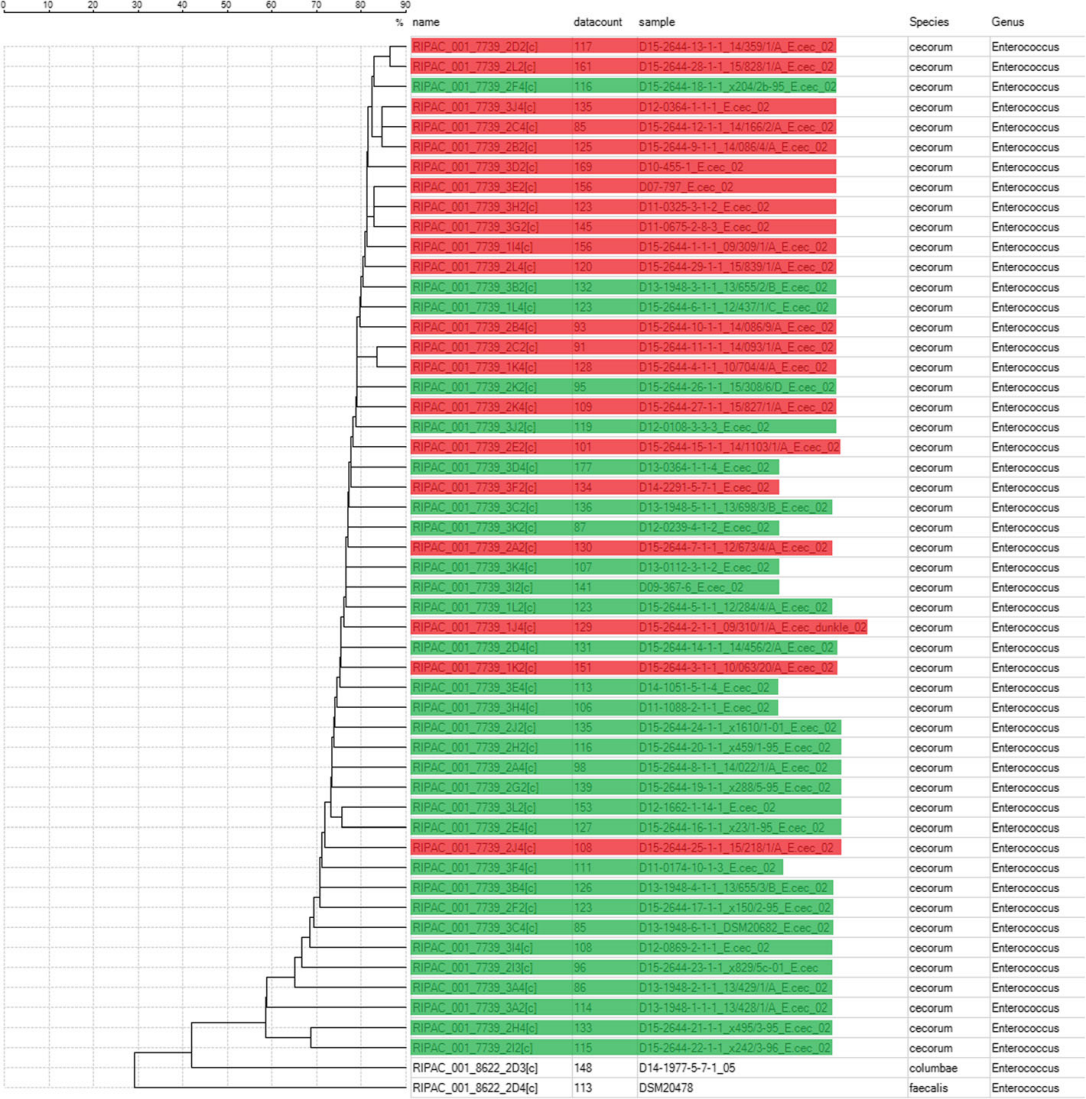
Dendrogram based on MALDI-TOF MS data of 50 EC isolates plus the reference strain. Pathogenic EC isolates are highlighted in *red*, commensal isolates are highlighted in *green*

All isolates were confirmed as EC using MALDI-TOF mass spectrometry. *Enterococcus columbae* and *Enterococcus faecalis* served as outgroups and were clearly separated from all EC strains. All EC isolates formed a single cluster, which contained only one small subcluster (Fig. 4). Pathogenic strains seem to concentrate in the upper half of the dendrogram and commensal strains in the lower part, but pathogenic and commensal strains were not clearly separated from each other. In total, 514 different masses were detected in the 51 EC strains with the MALDI-TOF MS. Single masses with potential differences in pathogenic and commensal EC strains were selected for further statistical analysis. Masses 3787, 3788, 3922, 4594, 6322, 6527, 7869, 7882 and 8246 were detected significantly (*P* ≤ 0.05; Fisher’s exact test) more often in pathogenic isolate, while masses 3105, 3908, 7562, 7771 and 8356 were significantly more often found in commensal isolates (data not shown).

The subcluster consisted of two small colony variant strains x242/3-96 from laying hen and x495/3-95 from swan. The strain 13/428/1/A with normal colony size from laying hen was also located separately from the main cluster (Fig. 4).

### Detection of the fatty acid composition by gas chromatography

**Fig. 5:**
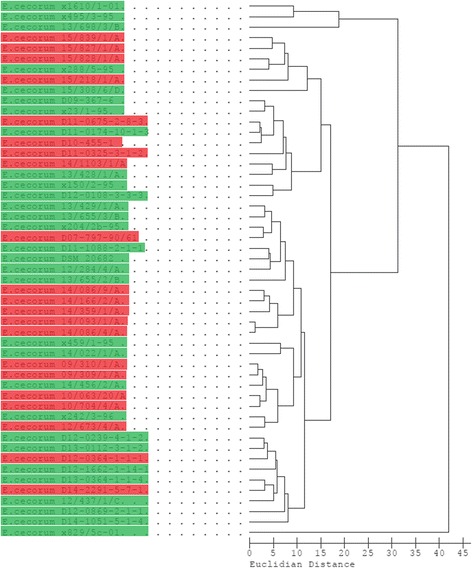
Dendrogram based on FAME profiles of 50 EC isolates plus the reference strain. Pathogenic EC isolates are highlighted in *red*, commensal isolates are highlighted in *green*

Dodecanoic acid (C_12:0_), tetradecanoic acid (C_14:0_), pentadecanoic acid (C_15:0_), hexadecanoic acid (C_16:0_), heptadecanoic acid (C_17:0_), octadecanoic acid (C_18:0_), (11Z)-11-octadecenoic acid (C_18:1_ w7c), (9Z)-9-octadecenoic acid (C_18:1_ w9c), (5Z,8Z,11Z,14Z)-5,8,11,14-eicosatetraenoic acid (C_20:4_ w6,9,12,15c), summed feature 3 (C_15:0_ iso 2OH and C_16:1_ w7c) and summed feature 5 (C_18:0_ anteiso and C_18:2_ w6,9c) were detected as the major fatty acids in the EC strains using gas chromatography (Table 3). Most of these fatty acids were detected in all EC strains, only C_12:0_, C_17:0_, C_18:1_ w7c and C_20:4_ w6,9,12,15c were missing in single isolates. In a dendrogram displaying the relatedness of the EC isolates based on their fatty acid profiles, no separate clustering of pathogenic and commensal isolates was found (Fig. 5). However, the dendrogram shows a clustering of EC strains isolated in 2015 from different disease outbreaks in broilers in northern Germany (15/839/1/A to 15/218/1/A). In all five strains, C_17:0_ anteiso and C_17:1_w8c were detected as additional fatty acids which were only demonstrated in 13 and 24 other EC strains respectively. Also, strains from broiler outbreaks in 2014 group together (14/086/9/A to 14/086/4/A). In all five of these strains, C_13:0_ was found, which was only detected in 21 of the other EC strains. Additionally, pathogenic isolates from duck and broiler outbreaks in 2009 and 2010 can be found in one separate cluster (09/310/1/A to 10/704/4/A). In all five isolates, C_20:1_ w7c was demonstrated, which was only found in 11 other EC strains. SCV strains x1610/1-01 and x495/3-95 formed a cluster together with the normal growing 13/698/3/B, but no cluster-specific FAME profile was recognizable. SCV strain x829/5c-01 at the bottom of the figure is separated from all other isolates with a Euclidean distance of over 40 units (Fig. 5). In contrast to all other EC strains, nonanoic acid (C_9:0_), decanoic acid (C_10:0_) and (12Z)-12-octadecenoic acid (C_18:1_ w6c) were detected in x829/5c-01, while (11Z)-11-octadecenoic acid (C_18:1_ w7c) was missing (data not shown).

**Table 3:** Cellular fatty acid composition of *Enterococcus cecorum* strains^a^

Major fatty acids^b^	Frequency (%)	Mean	Range
C_12:0_	92.2	0.6	0.0–1.5
C_14:0_	100.0	8.3	4.1–14.9
C_15:0_	100.0	2.6	0.9–4.4
C_16:0_	100.0	28.1	18.2–53.1
C_17:0_	98.0	0.9	0.0–1.9
C_18:0_	100.0	4.9	1.8–10.6
C_18:1_ w7c	98.0	33.1	0.0–46.9
C_18:1_ w9c	100.0	9.1	2.6–28.6
C_20:4_ w6,9,12,15c	70.6	0.4	0.0–1.7
Summed feature 3^c^	100.0	8.2	0.6–14.4
Summed feature 5^c^	100.0	2.4	1.4–7.8

^a^All 50 strains from this study plus the reference strain DSM 20682. ^b^For all other fatty acids, EC strains were negative or mean value was >0.27 and feature not used by MIDI system. ^c^Summed features represent groups of two fatty acids which could not be separated by gas chromatography with the MIDI system. Summed feature 3 contains C_15:0_ iso 2OH and C_16:1_ w7c. Summed feature 5 contains C_18:0_ anteiso and C_18:2_ w6,9c

### Mannitol metabolism

Only nine of the 50 examined EC isolates were able to utilize D-mannitol, and these were all commensal strains. None of the strains which were grouped into the pathogenic isolates were D-mannitol positive. Furthermore, all EC isolates from broilers, Pekin ducks and turkeys were mannitol negative, both pathogenic and commensal strains. D-mannitol positive isolates originated from laying hens, pigeons, cattle, swine and budgerigar.

### Serotyping

Nine of the isolates were classified as serotype 1, 13 as serotype 2 and 28 isolates were non-typeable with the two antisera (Table 4). None of the serotypes was predominantly found in the pathogenic or commensal isolates (*P* ≤ 0.05; Chi-Square test), the calculated *P* value was 0.574. The distribution of the serotypes among poultry species/production types were as follows: 6.7% of the broiler isolates, 9.1% of the Pekin duck isolates, 0% of the laying hen isolates, 40.0% of the turkey isolates and 75.0% of the pigeon isolates were classified as serotype 1. Additionally, 26.7% of the broiler isolates, 45.5% of the Pekin duck isolates, 16.7% of the laying hen isolates, 20.0% of the turkey isolates and 0% of the pigeon isolates were classified as serotype 2.

**Table 4:** Comparison of serotype prevalence in pathogenic and commensal *Enterococcus cecorum* strains

Serotype	Total no. of isolates	Pathogenic strains (*n* = 21)	Commensal strains (*n* = 29)
Serotype 1	9	3 (14.3%)	6 (20.7%)
Serotype 2	13	7 (33.3%)	6 (20.7%)
Non-typeable^a^	28	11 (52.4%)	17 (58.6%)

^a^With the two antisera which were produced for this study

### Detection of virulence factors

Genes for cytolysin (*cylA*) and cell wall adhesin of *E. faecium* (*efaAfm*) were not detected in any of the EC isolates. All other virulence genes were found at least in one isolate (Table 1). There was no predominating virulence factor found in pathogenic or commensal isolates (*P* ≤ 0.05; Fisher’s exact test). The most frequently detected gene was *gelE*, which was found in 8 strains, followed by *esp* in 6, *asa1* and *ccf* in 4 strains each and *hyl* in 3 strains. Gene *efaAfs* was found in only two strains.

### Chicken embryo lethality assay

The chicken embryo lethality of the examined EC isolates varied from 0 up to 100%. Most of the embryos died at days 2 and 3 post inoculation, but occasionally single embryos also died at days 4 to 7 post inoculation. The mean embryo lethality in pathogenic EC isolates was 39.7%, which was significantly (*P* ≤ 0.05; Kruskal-Wallis test, post hoc-test Dunn’s All-Pairwise Comparisons) higher than the mean embryo lethality of the commensal strains, which was 18.9%. The mean embryo lethality was 53.3% in swine, 45.4% in broiler, 43.4% in cattle, 40.0% in Muscovy duck, 22.3% in Pekin duck, 17.0% in laying hen, 16.0% in turkey, 13.3% in budgerigar, 13.3% in human, 1.7% in pigeon and 0.0% in swan isolates. Selected eggs were sampled for bacterial growth. EC was isolated from all tested eggs.

## Discussion

EC belongs to the physiological intestinal microbiota of chickens [28, 29] and probably also of other avian species. Nevertheless, EC is able to induce disease outbreaks not only in broilers, but also in Pekin ducks and sporadically in other bird species [14, 16–18]. There is not much knowledge about differences between intestinal isolates from healthy animals and isolates from diseased birds with pathologic changes. All data available were collected exclusively from EC strains isolated from broilers. In this study, we have compared both pathogenic and commensal isolates from 11 different animal species including one human isolate.

In a phylogenetic tree based on partial 16S-rRNA-gene no separate clustering of pathogenic and commensal EC strains was found. The segment of the 16S-rRNA gene which was used in this study was relatively short, therefore sequencing of the whole gene may lead to different results.

Lipids are important macromolecules which can be found mainly in the cytoplasmatic membrane and storage granules of bacteria and the cell wall of gram negative bacterial species. Fatty acids are the main component of these lipids. The fatty acid profile of bacteria can be detected and bacterial species differentiated using gas chromatography. In this study, we analyzed the fatty acid profile of all EC strains. We were able to show a set of 11 fatty acids which are typical for the majority of EC strains. To our best knowledge, this is the first report of the fatty acid composition of EC. No specific fatty acid profile could be found for either pathogenic or commensal isolates and strains of these categories formed no separate clusters in the dendrogram (Fig. 5). But remarkably, on a smaller scale, strains from EC disease outbreaks grouped clearly together, while in the dendrogam of the MALDI data, these strains were more randomly distributed (Figs. 4 and 5).

EC isolates from spinal lesions of diseased broilers showed a decreased ability to utilize D-mannitol in comparison with cecal isolates from healthy birds [44]. Additionally, genes which are involved in mannitol metabolism were found in non-pathogenic EC isolates, but were absent or probably non-functional in pathogenic strains [45]. The authors concluded that mannitol metabolism may be a useful marker for pathogenic EC strains, however, the role of mannitol in the infection is not known. In this study, only 9 of 29 commensal strains were mannitol positive, whereas all pathogenic isolates were mannitol negative. In fact, none of the intestinal isolates from broilers and Pekin ducks were mannitol positive, and these are the species/production types in which EC infections are most important. The reasons for the different results are unknown. However, Borst et al. used the Biolog system for metabolic profiling [44] while in this study a classical mannitol suspension in a test tube was used. Therefore, based on our results we conclude that if an EC strain is mannitol positive, it is probably a non-virulent isolate, but not vice versa.

In this study, different serotypes in EC were demonstrated for the first time. Two serotypes were differentiated by slide agglutination. However, the majority of the isolates were non-typeable with the two sera, indicating the existence of additional serotypes. No serotype was predominantly found in pathogenic or commensal strains. These results may change when further serotypes will be described. The demonstration of different serotypes of EC may be important regarding development and of vaccines and implementation of vaccine programs in poultry flocks as well as epidemiological investigations. Serotype 2 was more prevalent in broiler and Pekin duck isolates than serotype 1. However, serotype 1 dominated in the pigeon strains.

Very few *Enterococcus* virulence factors were detected in the majority of EC strains from North America and Poland [46, 47]. Also in our study, very few virulence factors were found in both pathogenic and commensal EC isolates from different animal species/production types via PCR (Table 5). No virulence factor was found significantly (*P* ≤ 0.05) more often in the pathogenic EC strains compared to the commensal isolates in our study. All data concerning virulence factors in EC collected so far indicate that virulence genes which are described in other *Enterococcus* species do not explain the ability of EC to induce severe disease. Recently, 3 pathogenic and 3 commensal EC strains were compared using whole genome sequencing [45]. Several unique genomic features were described in the pathogenic isolates, which have to be verified via sequencing of more EC isolates Using a chicken embryo lethality assay, pathogenic EC isolates induced significantly higher embryo lethality than commensal strains in this study. This property was already shown for broiler isolates [48], but never for EC strains from other animal species. Isolates from broilers, Muscovy duck and Pekin ducks, in which EC infection was reported, showed a relatively high embryo lethality whereas isolates from laying hens, turkeys and pigeons, in which disease due to EC are unknown or rarely seen, showed low embryo lethality. EC strains from cattle and swine represented an interesting exception with relatively high lethality rates in chicken embryos. However, there are no reports available of EC associated diseases in these species. In summary, we have done the first comparison of virulence of EC strains from different animal species. This technique can be used as a screening method for virulent strains, although the genetic background for higher virulence is still not fully understood.

**Table 5:** Comparison of virulence factor prevalence in pathogenic and commensal *Enterococcus cecorum* strains

Virulence gene^a^	Total no. of isolates	Pathogenic strains (*n* = 21)	Commensal strains (*n* = 29)	*P* value^b^
*cylA*	0	0 (0.0%)	0 (0.0%)	-^c^
*esp*	6	3 (14.3%)	3 (10.3%)	0.686
*hyl*	3	3 (14.3%)	0 (0.0%)	0.068
*asa1*	4	0 (0.0%)	4 (13.8%)	0.129
*gelE*	8	3 (14.3%)	5 (17.2%)	1.000
*efaAfm*	0	0 (0.0%)	0 (0.0%)	-^c^
*efaAfs*	2	1 (4.8%)	1 (3.4%)	1.000
*ccf*	4	1 (4.8%)	3 (10.3%)	0.630

^a^Corresponging virulence factors are listed in Table 2. ^b^A *P* value of *P* ≤ 0.05 was considered statistically significant by Fisher’s exact test. ^c^Not applicable

During production of subcultures of the strains in this study, we recognized two different growth patterns of the isolates. Most of the strains showed normal colony sizes with a diameter of approximately 2–3 mm after 24 h incubation, whereas a minority of strains developed only very small colonies with a diameter of less than 1 mm. After Gram staining, these strains consisted of cocci with very heterogeneous sizes and shapes, including conglomerates of large cocci. This appearance in Gram staining was also reported for *Enterococcus faecalis* small colony variant (SCV) strains from humans [42]. Interestingly, in each phylogenetic analysis of 16S-rRNA sequencing, MALDI-TOF MS and FAME profiles, at least a part of the SCV strains group separately from the main cluster. Therefore, we assume that SCV strains are phylogenetically distant from EC strains with normal colony morphology. SCV strains of *Enterococcus faecalis* appeared to be more virulent in a challenge model in laying hens where amyloid arthropathy was induced [43]. Our SCVs of EC demonstrated no higher virulence using the chicken embryo lethality assay and were all categorized as commensal and older strains isolated from 1995 to 2001. Nevertheless, this study represents the first description of SCV in EC.

This study describes the first comparison of different properties using EC isolates from different animal species, including pathogenic and commensal isolates. Furthermore, it is the first description of EC in the avian species budgerigar and swan and the first comparison of EC strains from animals and humans. Although the human EC strain was isolated from a hospitalized person with EC septicemia, it demonstrated only a low chicken embryo lethality of 13.3%. This result may be explained by the different host species of the test system. However, also no virulence factors were detectable via PCR, which shows how little we know about this bacterial species and illustrates the necessity of further research concerning epidemiology and pathogenesis of EC associated diseases.

## Conclusions

We compared pathogenic and commensal EC strains from different animal species. Pathogenic isolates showed higher chicken embryo lethality, the ability to metabolize mannitol and showed divergent mass peak patterns with MALDI-TOF MS. These differences may be explained by a separate evolution of pathogenic EC isolates. Additionally, different serotypes of EC were demonstrated, although no predominant serotype was found in pathogenic or commensal isolates. Our observations may be important for investigations of EC field strains or selection of isolates for the production of vaccines.

## Abbreviations

CSB agar: Columbia sheep blood agar
EC: *Enterococcus cecorum*
FAMEs: Fatty acid methyl esters
MALDI-TOF MS: Matrix-assisted laser desorption & ionization time-of-flight mass spectrometry
PCR: Polymerase chain reaction
SCV: Small colony variant
